# Exploration and validation of related hub gene expression during SARS-CoV-2 infection of human bronchial organoids

**DOI:** 10.1186/s40246-021-00316-5

**Published:** 2021-03-16

**Authors:** Ke-Ying Fang, Wen-Chao Cao, Tian-Ao Xie, Jie Lv, Jia-Xin Chen, Xun-Jie Cao, Zhong-Wei Li, Shu-Ting Deng, Xu-Guang Guo

**Affiliations:** 1 Department of Clinical Laboratory Medicine, The Third Affiliated Hospital of Guangzhou Medical University, Guangzhou, 510150 China; 2 Department of Clinical Medicine, The Third Clinical School of Guangzhou Medical University, Guangzhou, 511436 China; 3 Key Laboratory for Major Obstetric Diseases of Guangdong Province, The Third Affiliated Hospital of Guangzhou Medical University, Guangzhou, 510150 China; 4 Key Laboratory of Reproduction and Genetics of Guangdong Higher Education Institutes, The Third Affiliated Hospital of Guangzhou Medical University, Guangzhou, 510150 China

**Keywords:** Immune response, Human bronchial organoids, Novel coronavirus infection, Bioinformatics analysis, 3D structure model

## Abstract

**Background:**

In the novel coronavirus pandemic, the high infection rate and high mortality have seriously affected people’s health and social order. To better explore the infection mechanism and treatment, the three-dimensional structure of human bronchus has been employed in a better in-depth study on severe acute respiratory syndrome coronavirus 2 (SARS-CoV-2).

**Methods:**

We downloaded a separate microarray from the Integrated Gene Expression System (GEO) on a human bronchial organoids sample to identify differentially expressed genes (DEGS) and analyzed it with R software. After processing with R software, Gene Ontology (GO) and Kyoto PBMCs of Genes and Genomes (KEGG) were analyzed, while a protein–protein interaction (PPI) network was constructed to show the interactions and influence relationships between these differential genes. Finally, the selected highly connected genes, which are called hub genes, were verified in CytoHubba plug-in.

**Results:**

In this study, a total of 966 differentially expressed genes, including 490 upregulated genes and 476 downregulated genes were used. Analysis of GO and KEGG revealed that these differentially expressed genes were significantly enriched in pathways related to immune response and cytokines. We construct protein-protein interaction network and identify 10 hub genes, including IL6, MMP9, IL1B, CXCL8, ICAM1, FGF2, EGF, CXCL10, CCL2, CCL5, CXCL1, and FN1. Finally, with the help of GSE150728, we verified that CXCl1, CXCL8, CXCL10, CCL5, EGF differently expressed before and after SARS-CoV-2 infection in clinical patients.

**Conclusions:**

In this study, we used mRNA expression data from GSE150819 to preliminarily confirm the feasibility of hBO as an in vitro model to further study the pathogenesis and potential treatment of COVID-19. Moreover, based on the mRNA differentiated expression of this model, we found that CXCL8, CXCL10, and EGF are hub genes in the process of SARS-COV-2 infection, and we emphasized their key roles in SARS-CoV-2 infection. And we also suggested that further study of these hub genes may be beneficial to treatment, prognostic prediction of COVID-19.

## Introduction

In late December 2019, Wuhan, China, reported an epidemic situation of viral pneumonia caused by unknown microbial pathogens. Its clinical manifestations are very similar to those of viral pneumonia, including fever, cough, shortness of breath, myalgia, and fatigue [1].

On January 7, the pathogen, which was now named as SARS-CoV-2 [2], of this outbreak was identified by the Chinese Center for Disease Control and Prevention (CDC) from a throat swab sample of a patient [3].

SARS-CoV-2 is a new member of the Coronaviridae family [4]. Similar to other coronaviruses, the main routes of transmissions are respiratory droplets, respiratory secretions, and direct contact [5]. Later, it was reported that SARS-CoV-2 could be isolated from fecal swabs, which explains its rapid spread. The rapid spread and high mortality are responsible for the massive global outbreak followed by the outbreak in China. Based on the situation, the World Health Organization (WHO) declared the outbreak to be a public health emergency of international concern on January 31, 2020 [6]. As of October 15, 2020, there are a total of 38,394,169 confirmed SARS-CoV-2 cases worldwide, including 1,089,047 deaths [7].

Because the novel coronavirus has a huge impact on social order, the global economy and, especially, people’s health, a large number of experiments have been done to study the susceptibility and mechanism of SARS-CoV-2. Many infection models were used to study the reaction and immunity of SARS-CoV-2 infected bodies and these models played an important role in these studies [8–13]. However, all the commonly used models have a limitation. Either they are suboptimal models to represent human bodies, or they are limited in number [10, 11, 14].

In order to better understand the interaction between human body and different pathogens, some gene expression research conducted in vivo or in affected patients has established a three-dimensional structural organ model composed of human stem cells [15–17]. These models have reproduced various cell components and the morphology as well as an arrangement of each kind of cell. They are believed to help in understanding the path mechanism and developing drugs and vaccines [15, 18]. Such an advantaged model was also used to learn about SARS-CoV-2. By now, organoids of different apparatuses have been built and these organoids greatly improved researchers’ knowledge toward SARS-CoV-2. A study of intestinal tract organoids indicated that people get infected by SARS-CoV-2 through the intestinal tract, and studies of nervous system-related organoids may explain SARS-CoV-2-infected patients’ neurologic symptoms [8, 9]. All these studies showed that organoids could be good models to study SARS-CoV-2.

Therefore, we hope to explore the interaction between SARS-COV-2 and human body through the bioinformatics analysis of human bronchial organoids’ mRNA expression data from GSE150819 data set and preliminarily explore whether human bronchial organoids can be used as an in vitro model for the study of SARS-COV-2 infection.

## Materials and methods

### Gene expression datasets

We searched the GEO (https://www.ncbi.nlm.nih.gov/gds/) database to find relevant datasets for our study. Dataset GSE150819 contains genomic information of human bronchial organoids, which we are interested in. Among all samples in GSE150819, 6 samples are available for our study including 3 samples from SARS-CoV-2 infected organoids and 3 from uninfected organoids, and these 6 samples were all sequenced by GPL24676 Illumina nova seq 6000 (homo sapiens) platform. The sample source is bronchial organoid. The detailed information is listed in Table 1.

**Table 1:**
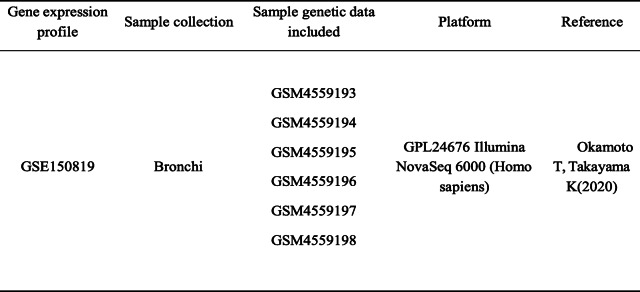
Details of the data sources for this study

### Identification of DEGs

After the gene expression data were obtained, we analyzed the data using the R package (R Foundation for Statistic Computing). Fold change (FC), *P* value, and false-positive rate were calculated to define DEGs between SARS-CoV-2 infected organoid samples and uninfected organoid samples. Finally, in the R language program, we selected the DEGs with logFC>1 (upregulated gene) or logFC<−1 (downregulated gene) calculated, while *P* < 0.05 was considered to be a significant difference and could be used to reduce the false-positive rate.

### Functional enrichment analysis of DEGs

The Gene Ontology (GO) database, containing three sections: biological progress (BP), cellular component (CC), molecular function (MF), and the Kyoto Encyclopedia of Genes and Genomes (KEGG) database are the most popular tools to learn about gene functions [19]. Therefore, two databases and the reference value *P* value < 0.05, which was considered as the significant difference, were used to further study the function of DEGs.

### PPI network construction and hub gene analysis

The Search Tool for the Retrieval of Interacting Genes/Proteins (STRING) (https://string-db.org/) is an online database collecting a large amount of protein interaction information. To learn about protein interaction, the STRING online tool was used. Protein interaction is admitted when the combined score was >0.4. We visualized the protein-protein interaction (PPI) network with the usage of Cytoscape software. To study protein interaction further, we used a plug-in to filter to hub genes. And they get the highest degree in the CytoHubba plug-in analysis, which means having the highest degree of connectivity within the PPI network [20].

### Validation of gene expression levels

We obtained the PPI network action diagram of differential gene expression, and screened out the central genes through the CytoHubba plug-in. These genes, as key genes, are the genes with the highest degree of connectivity or connecting multiple modules in the PPI network diagram. At the same time, we selected GSE150728 to detect peripheral blood mononuclear cells (PBMCs) and verify the expression of pivotal genes. The expression values of the corresponding central genes were found from the expression matrix and organized into a table, which was then produced into a verification map using the GraphPad Prism8 software.

## Results

### Identification of DEGs

**Fig. 1:**
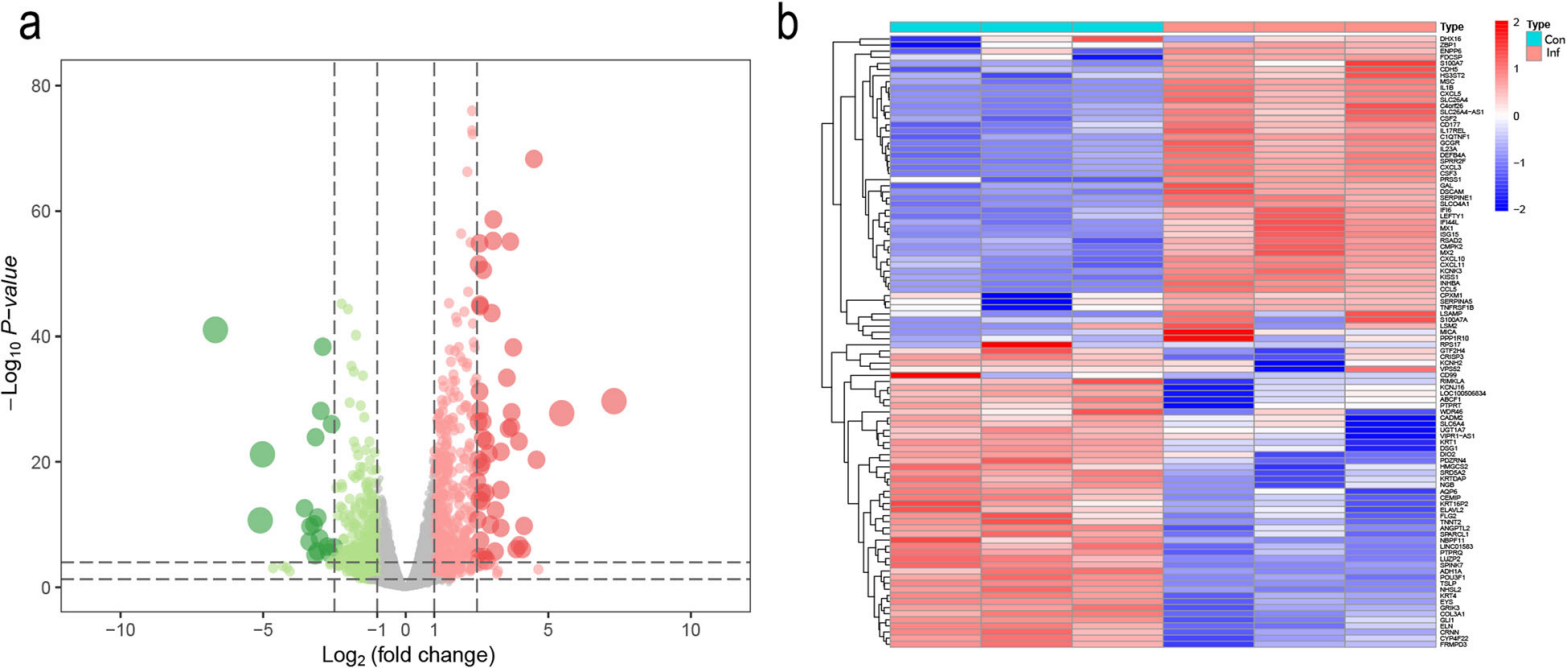
Heat map (**a**) and volcano plot (**b**) of the differentially expressed genes (DEGs). **a** Heat map of DEGs. The abscissa axis represents sample types and the ordinate axis represents gene names. **b** Volcanic plot analysis of upregulated genes and downregulated genes in infected and control groups. In total, 966 DEGs, including 490 upregulated and 476 downregulated DEGs, were identified in the GSE150819 when comparing the infected group and control group. Red indicates log_2_FC>1 while green indicates log_2_FC<−1, which means different gene expression between two groups (red for upregulated DEGs and green for downregulated DEGs). And gray indicates no difference

A data set (GSE150819) was obtained from the GEO database and analyzed in the R language. We found 966 DEGs between SARS-CoV-2 infected and uninfected group in total, among which 490 genes were upregulated and 476 genes were downregulated (Table 2). Volcano plot and heatmap of DEGs were also established. A volcanic map was generated to show up (red) and down (green) genes between infection and normal control (Fig. 1a). Afterwards, we used the heatmap package in R to plot a heatmap based on the expression levels of DEGs in GSE150819 (Fig. 1b).

**Table 2:** Upregulated genes and downregulated genes that meet the screening criteria

DEGs	Gene terms
Upregulated	KPNA7,RAB32,TMEM132A,CD7,A1BG-AS1,CDH2,ANGPTL4,BAMBI,ULBP1,DDX58,CYP8B1,CSTB,PNMA2,NEFM,SOCS1,NCF2,LINC00152,SOD2,THBS1,CYR61,HCG4B,IFITM2,COL6A2,TAGLN,TGFBR3L,TINAGL1,RASGRP3,TNFAIP2,ST6GALNAC5,GOLT1A,NALCN,TMIE,ABCA12,LRRC3,HMGA2,RELT,MTNR1A,CAPN14,ELOVL3,CEACAM19,GRAP,DGKI,NLRC4,BST2,DDIT4L,GBP4,HGD,ISG20,ZBED2,FEZ1,TMEM92,PRSS30P,FTH1,C10orf55,SIRPB2,MTHFD2,ARL4C,SUSD2,ITGB2,THY1,FUOM,RAB3B,KIF12,CBS,CPXM2,IFITM3,GAS6,LOC644936,MMD,LCP1,CTGF,FNDC4,EGOT,NRIP3,GFI1,WNT11,ARL9,ZC3H12A,MOGAT2,RCN3,IL2RG,MPV17L,TMEM106A,TMEM163,ADM2,LINC01215,FAP,SLC2A6,KRT7,HSD17B14,GREB1L,UCHL1,ALOXE3,NCF1,S100A12,RAC2,CYGB,ITGA5,NDP,PTAFR,NWD2,RFTN1,FAM155B,ALOX15B,REN,BATF2,PNMAL2,CPNE4,MEOX1,IRF7,ZNF876P,SLC34A2,HSH2D,GLDC,TNFSF14,SLC26A9,HDAC9,NKILA,RASGRF2,BIRC3,EGF,TUBB3,DPYSL4,DDX60,SLC8A2,FSTL3,ADAMTS1,SERPIND1,GSTM3,NFKBIA,TREM1,ADAM8,SCG5,SLC15A3,PARM1,DDO,RHEBL1,TSPAN2,CHD5,LHX5,FBLN7,PDCD1LG2,DUSP5,UCA1,C3,ARSF,BICC1,HIST1H1E,XKR9,PDZK1IP1,SAMD14,BCL2L15,IL24,GRIP2,FZD9,1-Dec,FGF2,MT1L,NOTCH4,IL7,KYNU,RBM24,TMPRSS2,TNIP1,THEMIS2,RENBP,FAM181B,VNN3,LOC101927865,LOC101927571,EDN1,RASD2,USP18,TNFAIP3,CYB5R2,CPA6,MAPK8IP2,SLC39A8,TYMP,DYSF,IER3,XAF1,CSF1R,OAS3,CAMK1G,CD83,IL15,LOC100130476,TRIM36,HKDC1,TAGLN3,RHCG,PDGFRL,PTP4A3,CILP2,ITPKA,LOC100505622,FABP6,PRRX2,CD200,BVES,TRIB3,KLHDC7B,IFI35,CST6,LOC102724279,RNU6-2,LAPTM5,SLC13A5,MFI2,BMP2,KRTAP2-3,KLK8,CCRL2,DHRS2,CD79A,SRGN,KLK9,PTPRH,ZFP42,CXCL6,ZG16B,WFDC5,HSD17B2,HBEGF,MUC3A,SEMA7A,PTGS2,PI3,MAFF,ADAMTS4,MEI1,GRB14,KMO,TMEM171,C6orf223,LCN2,HLA-B,KCNG1,LOC102467080,ATP6V0D2,IFI44,RPSAP52,GAS6-AS2,SERPINB7,OAS2,GPR176,TFPI2,CD70,GGT1,OTOP3,SHISA2,FN1,CTSE,DUOX2,LEMD1,NTM,KCCAT211,ANXA6,MIR146A,SH3RF3,RNASE1,PRSS22,LOC101929705,NODAL,LAMC2,IFIT2,ADAM19,INSC,C3orf70,IGFN1,DAAM2,CXCL1,NOG,HERC6,BPGM,HHIP,FAM169B,NEURL3,AMTN,IL6,PIWIL2,NCF1C,TBXAS1,FPR1,AANAT,PLAUR,CFB,TAF7L,BAG6,CDA,ASPHD1,LGALS2,TRABD2A,ICAM1,CCL2,ESM1,VNN1,4-Mar,KLK6,SLC7A7,HLA-F,NT5E,IL7R,POU2F2,POPDC3,C10orf10,SLC5A1,NOXO1,SELL,SSC4D,ZNF114,STC2,ART3,ALDOB,SAA2,PLAU,IL36G,BEST1,SPRR2D,FAM205A,PARVB,MUC13,SH2D2A,EPSTI1,LIF,TNFRSF9,NES,SAA4,ADRA1B,AWAT2,RASD1,ANXA2P3,ADAMTS9,MMP7,XDH,IL4I1,RAB42,PSG9,LAMP3,ANKRD1,HSD11B1,RND1,TNIP3,NOX1,ADAMTS6,UBD,IL1R2,MPZ,WNT7A,SV2B,IL32,MYCT1,MT2A,BCL2A1,DIO3,IDO2,TRPV2,NTNG1,LOC102723649,CXCL9,SNAI1,SYNGR3,IDO1,OLFM1,KLK5,KCNN3,RNASE7,C15orf48,SPRR2C,APOBEC3A,ROBO4,HEPHL1,SYNDIG1,HMGN2P46,SAA1,CUX2,IFITM1,SLC5A8,B4GALNT2,LINC00880,COL22A1,GFPT2,L1CAM,GDF15,CXCL8,MSC-AS1,IL19,PLA2G4C,TFF1,IFIT3,TRHDE,CXCL2,ATRNL1,CHAC1,G0S2,GBP5,CLLU1OS,SELP,C8orf4,MMP8,ZNF385C,MYH16,FOLR3,PDE4B,MRGPRX3,MMP9,SLC7A2,C19orf38,GRIN2A,MCHR1,PITX3,IFIT1,IL12RB1,COL13A1,IL17C,NCF1B,IL1A,CEACAM7,SMOC1,CCL20,SERPINA3,CD69,IFI27,ALPL,EPS8L3,OASL,LOC100126784,BEAN1,AMZ1,SPRR2A,SLC5A5,PDGFB,SPRR2E,HERC5,EBI3,CDH19,CYP27A1,DUOXA2,LOC101929427,SLC26A4-AS1,SERPINE1,MX1,ISG15,CXCL3,INHBA,HS3ST2,C4orf26,DSCAM,MSC,CD177,KISS1,SLCO4A1,C1QTNF1,CPXM1,MICA,IL17REL,LSAMP,IFI6,LEFTY1,IFI44L,SERPINA5,CXCL10,CXCL5,SPRR2F,S100A7A,GAL,KCNK3,VPS52,PPP1R10,GCGR,S100A7,CCL5,IL1B,IL23A,CMPK2,SLC26A4,MX2,CXCL11,DEFB4A,TNFRSF1B,RSAD2,ZBP1,ENPP6,CDH5,CSF3,CSF2,LSM2,FDCSP,PRSS1
Downregulated	KRT1,DIO2,DSG1,GTF2H4,RPS17,WDR46,DHX16,GRIK3,ABCF1,LUZP2,CYP4F22,UGT1A7,SPINK7,PDZRN4,ANGPTL2,KCNJ16,COL3A1,KRT4,LOC100506834,SRD5A2,CADM2,KCNH2,CRNN,AQP6,HMGCS2,GLI1,VIPR1-AS1,LINC01583,SPARCL1,SLC6A4,KRTDAP,PTPRT,FRMPD3,PTPRQ,TSLP,NHSL2,ADH1A,CEMIP,ELN,FLG2,KRT16P2,POU3F1,NBPF11,TNNT2,ELAVL2,EYS,NGB,RIMKLA,CRISP3,CD99,PLXDC1,EPB41L3,HMCN1,RPTN,APCDD1,AJAP1,IGFBP5,MAP7D2,SDR9C7,SNORD59B,PPP1R42,FAT3,PSAPL1,PRKCB,SLURP1,STEAP3-AS1,KRT15,EVPLL,BGN,ANPEP,POSTN,MPPED2,SMIM17,SLC10A5,EPHA3,SHISA9,C1orf168,SLC1A3,ASXL3,KRT13,RBM20,ANXA10,AADAC,FGF14-AS2,SBSN,MANEA-AS1,CACNA1D,PPP1R16B,RERG,IGFL1,KRT10,LOC101927023,CCDC136,DCLK1,ELFN1,CCDC73,AR,RTN4RL1,LOC730101,HOXA2,HYDIN2,IGFL2,TNNI2,HTR2B,GATA3-AS1,GLI2,SYNPO2,GRM4,HLF,FAM153A,KANTR,SLC24A3,GPR20,MSI1,DAPL1,FAM46C,CXCL14,PCDH18,CLDN8,GPC3,MESP1,CCDC3,DNAH6,SLC47A2,DNASE1L3,COL21A1,TPTE2P1,LINC01121,CYP4Z2P,RTCA-AS1,UMODL1,ANKH,P2RY1,GSTA2,RDH12,DMRT3,ZNF157,CHRNE,CNR1,LY6H,KRT16P3,EPGN,MIR4523,FABP4,TMPRSS11B,GUSBP9,LINC01116,EPHA7,C14orf132,MCM10,H19,FOXP2,APOBEC2,TP73,SMAD5-AS1,MIR4653,SPOCK2,SAMD5,SOSTDC1,MTUS2,TAS2R14,TMEM200A,SNX31,BEND5,DNAJC22,CLIC5,CYP2C8,IL2RB,C2orf73,SYT13,NKAPP1,CILP,TGM3,LOC200772,ARHGEF33,OR7E47P,FAM25A,COLCA1,MAP2K6,TMEM220-AS1,KRT6C,NUDT10,CXCL13,PAK7,PDE6A,EPPIN,HOPX,MFAP4,RNF222,CYP4B1,POU2AF1,SCN2B,MAP6,SEMA3G,PIK3C2G,LOC101929378,ECM2,MBNL1-AS1,LOC101927318,KIT,IL12RB2,SCN9A,PDE3B,KCNH4,SLAMF8,FOXI1,GOLGA8T,C1orf101,LINC01206,MIR27B,CLSTN2,SESN3,HERC2P3,SEMA5A,CLEC18A,ADGRL3,EDN2,FAIM2,CH25H,NLGN4X,ZNF233,DUSP9,DLK2,CTD-2201I18.1,UGT2A1,CDCA7,MIR6753,NEIL3,KNDC1,ERICH3,SEPP1,SOD3,ADGRF5,LINC01094,LOC100507283,MIR4517,GOLGA6L3,KIAA2022,TINCR,LOC100506127,HS3ST6,FOXN1,LOC100286922,LINC01132,ITGA10,PAQR5,RGAG4,GPR63,CPA4,TGFBR3,SPON2,PNPLA1,LOC441178,ATP8B4,EML6,CYP7B1,WSCD2,ACVR2B-AS1,AQP5,RGS22,LOC100129203,SKIDA1,PI16,LOC105373383,ENDOU,UGT2B28,METTL7A,SPTSSB,FKBP5,TET1,FETUB,ARHGAP28,CKAP2L,SNORD31,MPL,E2F2,SALRNA1,BMS1P5,PDGFD,ANXA2R,SNHG21,MIR99AHG,LOC100130093,EXO1,CARD18,UNC5C,BCL11B,DNAH9,CDHR3,C2orf71,CD36,SYT8,IGSF11,OPCML,INO80B,PKMYT1,SPOCK3,USP6,ADH7,MIR200B,LOC100128076,SLC25A34,HRASLS,DMRT2,SEPT5-GP1BB,CENPA,CYP4F3,LOC101929567,ECT2L,BAALC,CYP4F29P,LMO2,LEF1,DEPTOR,HSD17B13,FAM216B,ARHGAP11B,ALS2CR12,ADAD2,AGBL2,CARNS1,KIF11,F5,RGMA,PDE7B,CYP2C9,PALM,UGT2B17,FIBIN,COL5A3,KLHL31,VIPR1,CCDC62,HEY2,SUSD5,LOC100130417,LINC00683,SNHG19,MKI67,IL5RA,GAS1,ADAMTS5,MIR23B,TUB,MROH9,DEPDC7,OR7E91P,JMJD1C-AS1,TDRD1,AQP2,TEN1-CDK3,MIR186,COMP,ADAM23,LGI3,TOP2A,AADACP1,NOS2,SAXO2,ZRANB2-AS1,HEPACAM2,C1orf87,PCSK1,WNT3A,FLRT1,LMNB1,SEMA6D,HECW2,TREX2,LOC100507462,FOXD2,MKNK1-AS1,NWD1,PTGS1,TRPC6,GSTA1,GATA3,RGS4,IL6R,HSPB3,ARL14,FAM19A2,NDC80,CCR7,ACSS3,SPAG8,ITM2A,ZNF853,LOC100131655,GPR78,IL33,CYP4F12,EPHA4,BMP7,IGSF10,CFAP74,DTL,LOC339260,CCNE2,FAM131B,FGFR1,KCNJ5,AQP1,KIF26A,CHGB,TNNT3,C5,CUBN,NFIA,TFF3,PROC,ARHGEF26,TDRD6,CEP55,MBNL3,IL16,LOC100507006,KCNQ5,ZNF367,SNORA63,NPNT,CHRNA7,LINC01410,INHBB,KLLN,PLA2G4F,NCAPG,EGFL6,LGALS4,COL2A1,FZD1,MERTK,DNAAF1,LINC00664,FAM20A,DACT2,KC6,ENTPD8,UCP3,CASC5,EPHA10,RAET1E,ZNF608,AMH,LOC100129917,MYO3B,FAM181A-AS1,KCNAB3,VWA3B,CFAP43,SUSD4,WDR76,GATM,IP6K3,TCF4,FAM65B,LINC01481,DNAH3,KIF18B,POLQ,DLGAP5,STAG3L1,ZFHX4,ABLIM2,ABCA9,LHX6,ITGB7,LOC101928020,PDK4,CYP2W1,C6orf118,FILIP1,LOC100130705,LRGUK,PRUNE2,DTHD1

### Functional enrichment analysis of DEGs

**Fig. 2:**
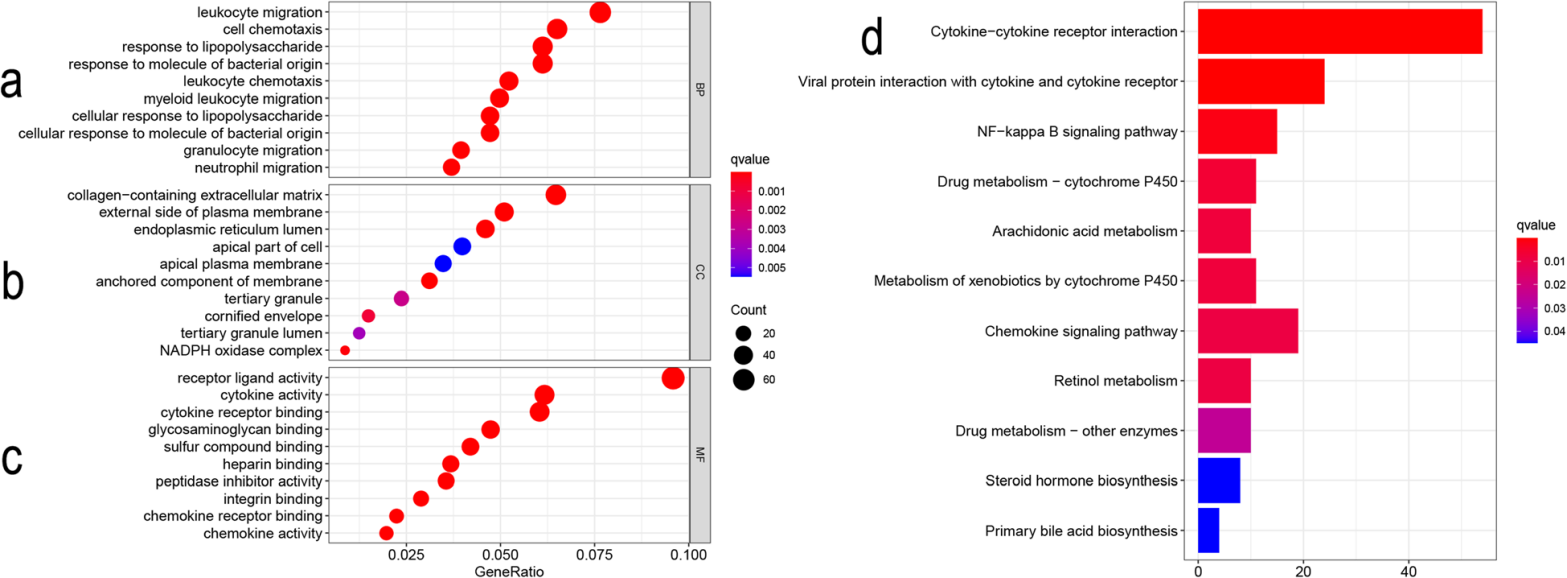
Bubble diagram results of GO analysis and histogram results of KEGG pathway analysis. Bubble diagram: The abscissa axis represents gene ratio while the ordinate axis represents term names. The size of a single bubble represents the degree of enrichment; the color variety represents different *q* value (those with red color are considered to be of significance). Histogram: The abscissa axis represents counts; the ordinate axis represents KEGG pathways; color represents the same meaning as the bubble diagram. **a** Top 10 enriched GO terms in biological process (BP); **b** top 10 enriched GO terms in cellular component (CC); **c** top 10 enriched GO terms in the molecular function (MF); **d** top 11 of enriched KEGG pathways

We analyzed the enrichment of DEGs, with two set databases GO and KEGG used. The first 10 biological processes of DEGs enrichment are shown in Fig. 2, including leukocyte migration, cell chemotaxis, and response to lipopolysaccharide (Fig. 2a). With regard to cellular components, the results showed that the collagen containing extra-cellular matrix was enriched, and the importance of extracellular matrix collagen components and plasma membrane in the progression of infection in the COVID-19 was gradually recognized (Fig. 2b). In addition, regarding the classification of molecular functions, DEGs focuses on the participation in the following functions: receptor–ligand activity, cytokine activity, and cytokine receptor binding (Fig. 2c). Importantly, KEGG analysis showed that the overlapping DEGs were mainly enriched in the cytokine receptor interaction and the viral protein interaction with cytokine and cytokine receptor (Fig. 2d). We described the difference in gene expression in the above pathway between the SARS-CoV-2 infected group and the uninfected group, respectively (Tables 3 and 4).

**Table 3:** The collection of significantly enricher GO pathways (top 10 according to *P* value)

Ontology	ID	Description	Count	*P* value	Gene ID
BP	GO:0060326	Cell chemotaxis	51	1.77E−17	IL1B/CCL20/CXCL8/CXCL5/SERPINE1/CXCL2/CXCL3/CXCL10/DEFB4A/CXCL1/IL23A/PDGFB/IL36G/SAA2/SAA1/CXCL11/RAC2/GAS6/CXCL6/HBEGF/CCL5/EDN1/ADAM8/SEMA5A/CXCL14/ITGB2/S100A7/THBS1/CXCL13/DYSF/IL6R/PDE4B/CH25H/IL16/KIT/EDN2/CXCL9/S100A12/CCL2/SAA4/TNFSF14/C5/FGFR1/LEF1/FGF2/CCRL2/PDGFD/CCR7/IL6/CYP7B1/SLAMF8
BP	GO:0030595	Leukocyte chemotaxis	41	1.21E−15	IL1B/CCL20/CXCL8/CXCL5/SERPINE1/CXCL2/CXCL3/CXCL10/CXCL1/IL23A/PDGFB/IL36G/SAA1/CXCL11/RAC2/GAS6/CXCL6/CCL5/EDN1/ADAM8/CXCL14/ITGB2/S100A7/THBS1/CXCL13/DYSF/IL6R/PDE4B/CH25H/IL16/KIT/EDN2/CXCL9/S100A12/CCL2/TNFSF14/C5/CCR7/IL6/CYP7B1/SLAMF8
BP	GO:0097529	Myeloid leukocyte migration	39	3.68E−15	IL1B/CCL20/CXCL8/CXCL5/SERPINE1/CXCL2/CXCL3/CXCL10/CXCL1/IL23A/PDGFB/IL36G/SAA1/CXCL11/RAC2/CXCL6/CCL5/EDN1/ADAM8/CD177/ITGB2/S100A7/THBS1/CXCL13/DYSF/IL6R/PDE4B/KIT/EDN2/CXCL9/S100A12/CCL2/C5/CD99/PDGFD/CCR7/IL6/CD200/SLAMF8
BP	GO:1990266	Neutrophil migration	29	6.55E−15	IL1B/CCL20/CXCL8/CXCL5/CXCL2/CXCL3/CXCL10/CXCL1/IL23A/IL36G/SAA1/CXCL11/RAC2/CXCL6/CCL5/EDN1/ADAM8/CD177/ITGB2/CXCL13/DYSF/PDE4B/EDN2/CXCL9/S100A12/CCL2/CD99/CCR7/SLAMF8
BP	GO:0097530	Granulocyte migration	31	2.10E−14	IL1B/CCL20/CXCL8/CXCL5/CXCL2/CXCL3/CXCL10/CXCL1/IL23A/IL36G/SAA1/CXCL11/RAC2/CXCL6/CCL5/EDN1/ADAM8/CD177/ITGB2/S100A7/THBS1/CXCL13/DYSF/PDE4B/EDN2/CXCL9/S100A12/CCL2/CD99/CCR7/SLAMF8
BP	GO:0032496	Response to lipopolysaccharide	48	4.74E−14	IL1B/CSF3/CXCL8/CXCL5/SERPINE1/CXCL2/CXCL3/CXCL10/ALPL/CXCL1/IDO1/TNFAIP3/IL36G/CXCL11/CXCL6/ICAM1/NFKBIA/CMPK2/PTGS2/SPON2/LCN2/ZC3H12A/CCL5/PTAFR/CSF2/GGT1/TNIP3/EDN1/ANKRD1/S100A7/CXCL13/PDCD1LG2/NOS2/PDE4B/KMO/CD36/TNFRSF1B/IL12RB2/CXCL9/CCL2/SELP/MIR146A/GFI1/CCR7/IL6/REN/CNR1/IL24
BP	GO:0071222	Cellular response to lipopolysaccharide	37	4.80E−14	IL1B/CSF3/CXCL8/CXCL5/SERPINE1/CXCL2/CXCL3/CXCL10/CXCL1/TNFAIP3/IL36G/CXCL11/CXCL6/ICAM1/NFKBIA/CMPK2/SPON2/LCN2/ZC3H12A/CCL5/PTAFR/CSF2/TNIP3/ANKRD1/CXCL13/PDCD1LG2/NOS2/PDE4B/KMO/CD36/TNFRSF1B/CXCL9/CCL2/MIR146A/GFI1/IL6/IL24
BP	GO:0071219	Cellular response to molecule of bacterial origin	37	1.42E−13	IL1B/CSF3/CXCL8/CXCL5/SERPINE1/CXCL2/CXCL3/CXCL10/CXCL1/TNFAIP3/IL36G/CXCL11/CXCL6/ICAM1/NFKBIA/CMPK2/SPON2/LCN2/ZC3H12A/CCL5/PTAFR/CSF2/TNIP3/ANKRD1/CXCL13/PDCD1LG2/NOS2/PDE4B/KMO/CD36/TNFRSF1B/CXCL9/CCL2/MIR146A/GFI1/IL6/IL24
BP	GO:0050900	Leukocyte migration	60	1.91E−13	IL1B/CCL20/CXCL8/CXCL5/SERPINE1/CXCL2/CXCL3/CXCL10/CXCL1/IL23A/PDGFB/IL36G/L1CAM/SAA1/CXCL11/RAC2/ITGA5/GAS6/CXCL6/ICAM1/CCL5/PTAFR/EDN1/SLC7A7/ADAM8/CD177/CXCL14/GATA3/FN1/IL33/ITGB2/S100A7/SELL/THBS1/CXCL13/DYSF/IL6R/PDE4B/CH25H/IL16/KIT/EDN2/CXCL9/ITGB7/S100A12/CCL2/MERTK/TNFSF14/THY1/C5/GRB14/TREM1/SELP/CD99/PDGFD/CCR7/IL6/CD200/CYP7B1/SLAMF8
BP	GO:0002237	Response to molecule of bacterial origin	48	2.08E−13	IL1B/CSF3/CXCL8/CXCL5/SERPINE1/CXCL2/CXCL3/CXCL10/ALPL/CXCL1/IDO1/TNFAIP3/IL36G/CXCL11/CXCL6/ICAM1/NFKBIA/CMPK2/PTGS2/SPON2/LCN2/ZC3H12A/CCL5/PTAFR/CSF2/GGT1/TNIP3/EDN1/ANKRD1/S100A7/CXCL13/PDCD1LG2/NOS2/PDE4B/KMO/CD36/TNFRSF1B/IL12RB2/CXCL9/CCL2/SELP/MIR146A/GFI1/CCR7/IL6/REN/CNR1/IL24
CC	GO:0062023	Collagen-containing extracellular matrix	52	2.39E−13	SERPINE1/ADAM19/HMCN1/SERPINA3/KRT1/GDF15/PDGFB/PRSS1/L1CAM/COL3A1/MMP9/ICAM1/SEMA7A/CSTB/LAMC2/GPC3/NPNT/SMOC1/TINAGL1/ANXA6/FN1/ELN/BMP7/COL21A1/ANGPTL2/EYS/COMP/ADAMTS1/EGFL6/SERPINA5/S100A7/CDH2/POSTN/THBS1/IL7/AMTN/CILP/COL5A3/COL13A1/ADAMTS5/BGN/COL6A2/SPARCL1/ADAMTS9/MMP8/MFAP4/SOD3/ADAMTS4/NDP/ECM2/LGALS4/COL2A1
CC	GO:0005788	Endoplasmic reticulum lumen	37	4.67E−09	WNT7A/PLAUR/COL22A1/IL23A/PDGFB/IGFBP5/COL3A1/GAS6/STC2/C3/PTGS2/FSTL3/TMEM132A/EBI3/GPC3/EDN1/FN1/COL21A1/PROC/CDH2/THBS1/AMTN/COL5A3/COL13A1/RCN3/ADAMTS5/COL6A2/SPARCL1/F5/ARSF/WNT3A/CYP2W1/PDGFD/CHGB/SERPIND1/IL6/COL2A1
CC	GO:0031225	Anchored component of membrane	25	2.69E−08	CEACAM7/ALPL/PLAUR/NT5E/PRSS22/SEMA7A/NTM/VNN1/GPC3/CD177/VNN3/RGMA/OPCML/BST2/ENPP6/FOLR3/NTNG1/GAS1/THY1/RAB3B/LSAMP/ART3/ULBP1/RTN4RL1/LY6H
CC	GO:0009897	External side of plasma membrane	41	3.80E−08	CXCL10/ITGA5/ICAM1/SEMA7A/EBI3/HLA-B/SSC4D/CD83/IL12RB1/SERPINA5/ITGB2/KCNJ5/CDH5/THBS1/IL6R/RAET1E/IL7R/HLA-F/PDCD1LG2/TNFRSF9/CD69/CD36/KIT/TGFBR3/IL12RB2/CXCL9/FOLR3/ANPEP/MICA/IL2RG/IL2RB/THY1/CUBN/SELP/IL5RA/ULBP1/RTN4RL1/CCRL2/CD79A/UMODL1/CCR7
CC	GO:0043020	NADPH oxidase complex	7	8.82E−07	DUOX2/NCF2/NOXO1/NOX1/NCF1B/NCF1/NCF1C
CC	GO:0001533	Cornified envelope	12	1.07E−05	SPRR2F/SPRR2D/KRT1/KRT10/SPRR2A/CST6/DSG1/PI3/SPRR2E/FLG2/RPTN/EVPLL
CC	GO:0070820	Tertiary granule	19	4.14E−05	CXCL1/PLAU/MMP9/CSTB/DSG1/PTAFR/CDA/ADAM8/CD177/FTH1/METTL7A/ITGB2/FLG2/FOLR3/FRMPD3/CRISP3/MMP8/ATP8B4/FPR1
CC	GO:1904724	Tertiary granule lumen	10	6.64E−05	CXCL1/MMP9/CSTB/CDA/FTH1/METTL7A/FLG2/FOLR3/CRISP3/MMP8
CC	GO:0045177	Apical part of cell	32	0.000111784	CEACAM7/SLC26A4/DUOXA2/SLC5A1/RHCG/DUOX2/KISS1/DSG1/PTPRH/AQP5/SLC34A2/FN1/P2RY1/AJAP1/SLC26A9/CDH2/SLC5A8/MUC13/BST2/CLIC5/IL6R/CYP4F12/AQP6/CD36/FAP/THY1/ATP6V0D2/CUBN/AQP2/ADGRF5/AQP1/REN
CC	GO:0016324	Apical plasma membrane	28	0.000117339	CEACAM7/SLC26A4/SLC5A1/RHCG/DUOX2/KISS1/DSG1/PTPRH/AQP5/SLC34A2/FN1/P2RY1/AJAP1/SLC26A9/CDH2/SLC5A8/MUC13/BST2/CLIC5/IL6R/CYP4F12/AQP6/CD36/THY1/ATP6V0D2/CUBN/AQP2/AQP1
MF	GO:0048018	Receptor ligand activity	73	2.69E−21	IL1B/IL1A/CCL20/CSF3/CXCL8/CXCL5/CXCL2/TSLP/CXCL3/INHBA/CXCL10/LIF/DEFB4A/CXCL1/WNT7A/GDF15/IL23A/PDGFB/IL36G/SAA2/TYMP/SAA1/CXCL11/GAS6/CXCL6/SEMA7A/STC2/BMP2/HBEGF/CCL5/IL32/EBI3/CSF2/EDN1/SEMA5A/CXCL14/IL19/BMP7/IL33/ADM2/IL17C/CXCL13/IL6R/EGF/TFF1/IL15/IL7/IL16/SEMA6D/EDN2/GAL/CXCL9/CCL2/EPGN/CD70/SAA4/SLURP1/LEFTY1/TNFSF14/SEMA3G/INHBB/C5/FGF2/WNT3A/PDGFD/CHGB/EPHA7/NDP/IL6/ENDOU/AMH/IL24/NODAL
MF	GO:0005125	Cytokine activity	47	3.18E−20	IL1B/IL1A/CCL20/CSF3/CXCL8/CXCL5/CXCL2/TSLP/CXCL3/INHBA/CXCL10/LIF/CXCL1/WNT7A/GDF15/IL23A/IL36G/CXCL11/CXCL6/BMP2/CCL5/IL32/EBI3/CSF2/EDN1/CXCL14/IL19/BMP7/IL33/IL17C/CXCL13/IL15/IL7/IL16/CXCL9/CCL2/CD70/SLURP1/LEFTY1/TNFSF14/INHBB/C5/FGF2/NDP/IL6/IL24/NODAL
MF	GO:0005126	Cytokine receptor binding	46	8.30E−15	IL1B/IL1A/CCL20/CSF3/CXCL8/CXCL5/CXCL2/TSLP/CXCL3/INHBA/CXCL10/LIF/DEFB4A/CXCL1/GDF15/IL23A/IL36G/CXCL11/ITGA5/CXCL6/BMP2/CCL5/EBI3/CSF2/CXCL14/GATA3/BMP7/IL12RB1/CDH5/BAMBI/CXCL13/IL6R/IL15/IL7/TGFBR3/CXCL9/CCL2/CD70/LEFTY1/TNFSF14/INHBB/C5/CCRL2/IL6/AMH/NODAL
MF	GO:0005539	Glycosaminoglycan binding	36	1.43E−11	CXCL10/MMP7/SAA1/CXCL11/CXCL6/HBEGF/LAMC2/RNASE7/CEMIP/SEMA5A/SPOCK3/ANXA6/FN1/BMP7/COMP/ADAMTS1/SERPINA5/SELL/POSTN/THBS1/CXCL13/COL5A3/COL13A1/TGFBR3/ADAMTS5/BGN/FGFR1/SPOCK2/SELP/FGF2/RTN4RL1/FBLN7/SOD3/ECM2/SERPIND1/SUSD5
MF	GO:0008201	Heparin binding	28	7.83E−10	CXCL10/MMP7/SAA1/CXCL11/CXCL6/HBEGF/LAMC2/FN1/BMP7/COMP/ADAMTS1/SERPINA5/SELL/POSTN/THBS1/CXCL13/COL5A3/COL13A1/TGFBR3/ADAMTS5/FGFR1/SELP/FGF2/RTN4RL1/FBLN7/SOD3/ECM2/SERPIND1
MF	GO:0008009	Chemokine activity	15	1.11E−09	CCL20/CXCL8/CXCL5/CXCL2/CXCL3/CXCL10/CXCL1/CXCL11/CXCL6/CCL5/CXCL14/CXCL13/CXCL9/CCL2/C5
MF	GO:0042379	Chemokine receptor binding	17	1.71E−09	CCL20/CXCL8/CXCL5/CXCL2/CXCL3/CXCL10/DEFB4A/CXCL1/CXCL11/CXCL6/CCL5/CXCL14/CXCL13/CXCL9/CCL2/C5/CCRL2
MF	GO:0030414	Peptidase inhibitor activity	27	1.83E−08	SERPINE1/SERPINA3/GAS6/CST6/C3/SPINK7/CSTB/PI3/TFPI2/GPC3/SPOCK3/SERPINB7/BIRC3/WFDC5/SERPINA5/BST2/FETUB/TNFSF14/RENBP/C5/LEF1/SPOCK2/UMODL1/CARD18/SERPIND1/PI16/EPPIN
MF	GO:1901681	Sulfur compound binding	32	3.47E−08	CXCL10/MMP7/SAA1/CXCL11/CXCL6/HBEGF/LAMC2/SEMA5A/ANXA6/GSTM3/FN1/BMP7/COMP/ADAMTS1/SERPINA5/SELL/POSTN/CBS/THBS1/CXCL13/COL5A3/COL13A1/TGFBR3/ADAMTS5/FGFR1/SELP/FGF2/RTN4RL1/FBLN7/SOD3/ECM2/SERPIND1
MF	GO:0005178	Integrin binding	22	4.57E−08	IL1B/ISG15/COL3A1/ITGA5/ICAM1/SEMA7A/NPNT/CD177/FN1/COMP/EGFL6/ITGB2/THBS1/ITGB7/ADAMTS5/FAP/THY1/LCP1/ESM1/FGF2/ECM2/ADAM23

**Table 4:** Eleven most significantly enriched KEGG pathways

ID	Description	Count	***P*** value	P. adjust	Gene ID
hsa04060	Cytokine-cytokine receptor interaction	54	3.59E−20	3.41E−18	IL1B/IL1A/CCL20/CSF3/CXCL8/CXCL5/CXCL2/TSLP/CXCL3/INHBA/CXCL10/LIF/CXCL1/GDF15/IL23A/IL36G/CXCL11/CXCL6/BMP2/CCL5/IL32/CSF2/CXCL14/IL19/BMP7/IL33/RELT/IL12RB1/IL17C/CXCL13/IL6R/IL7R/TNFRSF9/IL15/IL7/IL16/TNFRSF1B/IL12RB2/CXCL9/CSF1R/CCL2/CD70/IL2RG/IL2RB/TNFSF14/INHBB/IL1R2/IL5RA/CCR7/IL6/AMH/IL24/NODAL/MPL
hsa04061	Viral protein interaction with cytokine and cytokine receptor	24	6.71E−12	3.19E−10	CCL20/CXCL8/CXCL5/CXCL2/CXCL3/CXCL10/CXCL1/CXCL11/CXCL6/CCL5/CXCL14/IL19/CXCL13/IL6R/TNFRSF1B/CXCL9/CSF1R/CCL2/IL2RG/IL2RB/TNFSF14/CCR7/IL6/IL24
hsa04064	NF-kappa B signaling pathway	15	5.89E−05	0.00186373	IL1B/CXCL8/CXCL2/CXCL3/CXCL1/PLAU/TNFAIP3/BCL2A1/ICAM1/NFKBIA/PTGS2/BIRC3/PRKCB/DDX58/TNFSF14
hsa00982	Drug metabolism—cytochrome P450	11	0.000306597	0.007281679	ADH7/GSTM3/GSTA2/GSTA1/CYP2C9/UGT1A7/ADH1A/UGT2A1/CYP2C8/UGT2B28/UGT2B17
hsa00590	Arachidonic acid metabolism	10	0.000471477	0.008885837	PTGS2/GGT1/ALOX15B/PLA2G4C/CYP4F3/PLA2G4F/PTGS1/TBXAS1/CYP2C9/CYP2C8
hsa00980	Metabolism of xenobiotics by cytochrome P450	11	0.000561211	0.008885837	ADH7/GSTM3/GSTA2/GSTA1/CYP2C9/UGT1A7/ADH1A/HSD11B1/UGT2A1/UGT2B28/UGT2B17
hsa04062	Chemokine signaling pathway	19	0.000840976	0.010436507	CCL20/CXCL8/CXCL5/CXCL2/CXCL3/CXCL10/CXCL1/CXCL11/RAC2/CXCL6/NFKBIA/CCL5/CXCL14/PRKCB/CXCL13/CXCL9/CCL2/NCF1/CCR7
hsa00830	Retinol metabolism	10	0.000878864	0.010436507	ADH7/AWAT2/CYP2C9/UGT1A7/RDH12/ADH1A/UGT2A1/CYP2C8/UGT2B28/UGT2B17
hsa00983	Drug metabolism—other enzymes	10	0.002812844	0.029691134	XDH/TYMP/CDA/GSTM3/GSTA2/GSTA1/UGT1A7/UGT2A1/UGT2B28/UGT2B17
hsa00140	Steroid hormone biosynthesis	8	0.005895127	0.053494911	HSD17B2/SRD5A2/UGT1A7/HSD11B1/UGT2A1/UGT2B28/UGT2B17/CYP7B1
hsa00120	Primary bile acid biosynthesis	4	0.006194148	0.053494911	CYP27A1/CH25H/CYP7B1/CYP8B1

### PPI network construction and hub gene analysis

**Fig. 3:**
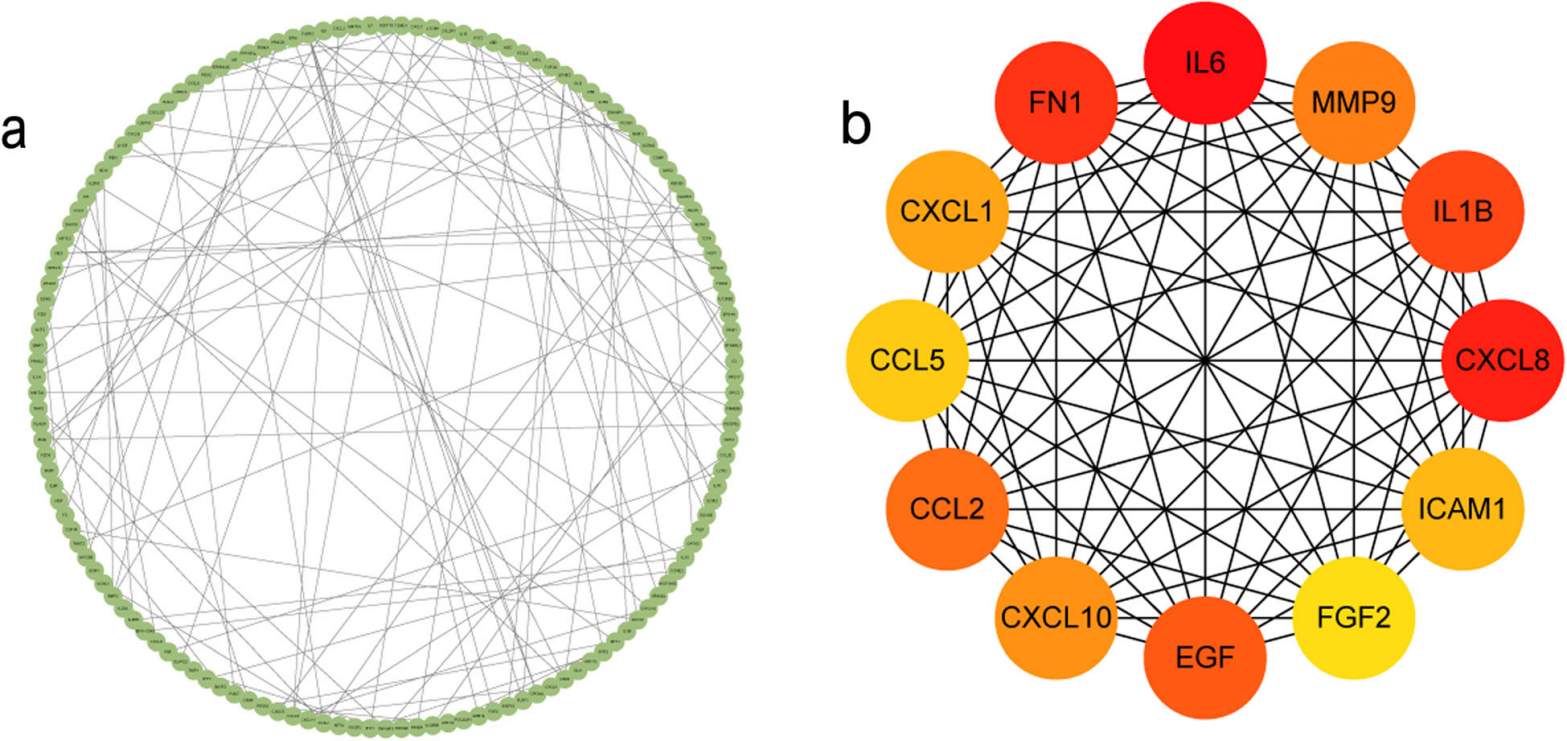
Protein–protein interactions (PPI) network, module analysis, and hub gene identification. The most significant modules were screened out by using the CytoHubba plug-in. Nodes represent gene; lines represent interactions between gene-encoded protein. **a** Construction of PPI networks. Direct molecular interaction network between novel coronavirus differently regulated genes supported by experiments, encoded protein; **b** PPI network of the top 12 hub genes (the redder the color, the more important it is)

We finally established the PPI network (Fig. 3a) through the STRING online tool and Cytoscape [21]. The network provides a clear and direct expression of complex interactions between gene node, and many domain analyses are based on network analysis. There are 140 nodes and 105 edges in the PPI network. We calculated the degree with the help of the CytoHubba plug-in. By doing so, we found out 12 hub genes and showed their interaction (Fig. 3b).

### Electronic validation of hub gene by GSE150728

**Fig. 4:**
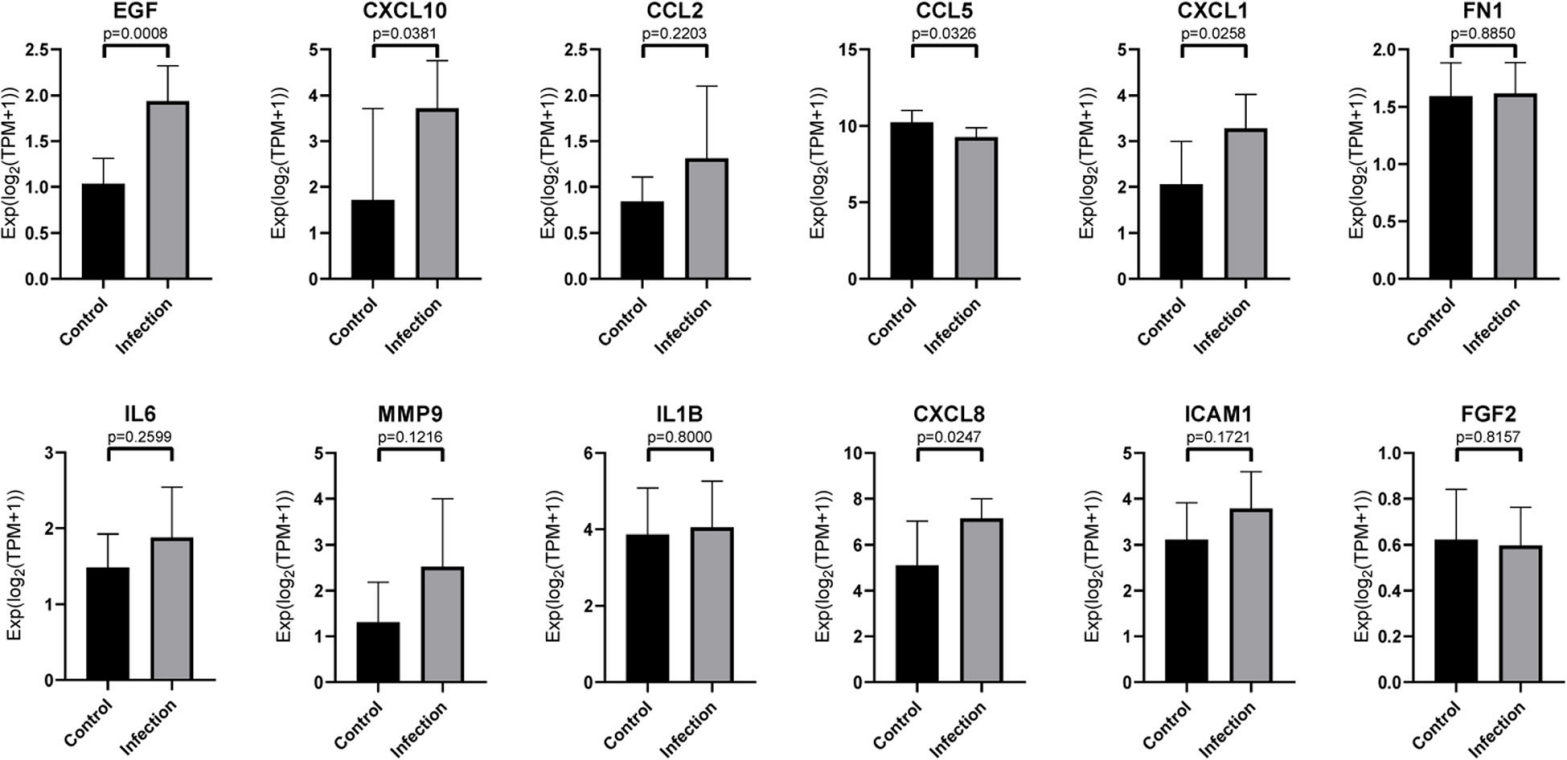
Validation of hub gene expression level. Genes are the top 12 selected from DEGs and PPI networks co-regulated by control and infected samples

GSE150728, performed on peripheral blood mononuclear cells (PBMCs), was selected to validate the expression of hub gene. In GSE150728 database, CXCL1, EGF, CXCL10, CXCL8, and CCL5 were differently expressed before and after SARS-CoV-2 infection. Among these genes, CCL5 was the only gene, whose levels decreased. Anyhow the electronic validation support the finding that CXCL1, EGF, CXCL10, and CXCL8 were significantly expressed in SARS-CoV-2 infected bronchial organoid when compared to normal bronchial organoid (Fig. 4)

## Discussion

Pneumonia caused by an infection in novel coronavirus has become a popular public health crisis in the world [22]. Its early diagnosis and treatment are crucial for later disease progression. In this paper, the related molecular and infectious mechanisms of the human bronchus-like tube after its infection with novel coronavirus were discussed from the perspective of bioinformatics analysis.

In this study, we analyzed six samples from the GSE150819GEO database and found 966 differential genes, of which 490 were upregulated and 476 were downregulated, resulting in the establishment of a volcanic map. In addition, we performed GO and KEGG analyses on the dataset. Among them, biological processes are highly enriched in molecules with leukocyte migration, cell chemotaxis, response to lipopolysaccharide, and bacterial origin, which may be related to an inflammatory reaction [23–25]. In the inflammatory response, cytokines secreted by endothelial cells react with leukocyte ligands to express adhesion molecules, chemokines, and chemoattractants. Chemokines bind to glycosaminoglycans on the surface of epithelial cells, triggering leukocyte recruitment and inducing integrin activation. Second, leukocytes also enter the basement membrane and migrate to the mesenchyme, causing significant enrichment and expression in the collagen in the extracellular matrix, the outside of the plasma membrane, and the endoplasmic reticulum lumen of the cell components, which is directly related to the mechanism of pneumonia caused by the novel coronavirus [26]. In the KEGG analysis, the enrichment of cytokine–cytokine receptor interactions and chemokine signaling pathways correspond to the enrichment of cellular components and molecular functions. The high aggregation of the interaction of viral proteins with cytokines and cytokine receptors suggested that viral proteins might be involved in related processes such as inflammation factor storm and inflammation factor regulation. The expression of endoplasmic reticulum collagen in cellular components also suggested that viral proteins might affect the transport process of endoplasmic reticulum. As a pathway to promote cell function, NF−kappa B signaling pathways activation may be related to the synthesis of viral proteins. In summary, the potential host-virus protein interactions may provide us with new ideas and strategies for drug screening and the development of drug-related research in SARS-CoV-2 [27]. At the same time, based on the PPI network diagram, we used the CytoHubba plug-in to select 12 central genes with high connectivity, all of which occupied the core nodes of the network, suggesting that these central genes might play a pivotal role in the process of infection in the novel coronavirus [20].

There are several highly interacting genes in the PPI network, such as CXCL1, EGF, CXCL10, CXCL8, CCL5, and what calls for special attention is that CCL5 levels decreased in peripheral blood mononuclear cells (PBMCs), whose data is form GSE150728, the dataset we used for electronic validation of hub gene. However, CCL5 was repeatedly reported to be increased in patients infected by SARS-CoV-2 [28], which supported the result we get from hBO. The unique inflammatory chemokine CCL5 (RANTES) induced at a later phase of inflammation guides white blood cells to migrate into inflammatory lesions during varied pathological processes and maintains local immune cells [29]. It has been demonstrated that CCL5 functions through activating the pathways including transcription factor 3 signaling transducers and activators, nuclear factor-κb, and MAPK pathways across three cell surface receptors named CCR1, CCR3, and CCR5. In addition, CCL5 can directly activate M1 (pro-inflammatory) polarization and prevent M2 (anti-inflammatory) polarization [30]. The lack of homeostatic CCL5 expression will largely influence the activated state of lung TRM (tissue-resident memory) T cell and natural killer cell components [31].

The chemokine CXCL1 plays a significant role in the immune response, mediating its function by combining with the CXCR2 receptor and GAG [32]. It highly regulates the transport, collection, and activation of neutrophilic granulocyte [33]. What is more, it activates the release of proteases as well as reactive oxygen species (ROS), killing the microorganisms at tissue [32]. But it is also associated with damage in numerous tissues, including the lung [34].

Data from previous coronavirus prevalence, including severe acute respiratory syndrome as well as the Middle East respiratory, and current data from the COVID-19 pandemic indicated that there could be pulmonary fibrosis after SARS-CoV-2 infection [35]. EGF may play an important role in pulmonary fibrosis after SARS-CoV-2 infection. Studies of SARS-CoV and SARS showed that abnormal expressions of EGFR are vital in the pathogenesis of fibrosis during coronavirus infection [36, 37]. An animal study also reported EGFR potential role in enhancing disease when infected by SARS-CoV [37]. So, it is of great significance to study EGF function in SARS-CoV-2 infected body, which may help prevent pulmonary fibrosis and learn further about the pathogenesis of SARS-CoV-2 induced lung injury.

CXCL10, an interferon-inducible protein, increases greatly in SARS-CoV-2 infected patients [38, 39]. Abnormal increase of CXCL10 abnormal is also incurred in SARS infected patients and indicates the worse clinical outcome [40, 41]. A study conducted on mice showed that CXCL10 may be involved in fulminant lung injury which can be modified when CXCL10 was neutralized [42]. Back to SARS-CoV-2, serum concentrations of CXCL10, combining with GM-CSF and SARS-CoV-2 viral load are associated with day-28 mortality [43]. All these indicated that CXCL10 can be a potential indicator and a therapeutic target for SARS-CoV-2.

In our study, increased expressions of CXCL10 and CXCL8 are observed, which also seem to characterize COVID-19. CXCL8 (IL-8) is a member of the chemokine family, and its receptors are CXCR1/2 [41]. The combination of CXCL8 and CXCR1 or CXCR2 will lead to lung injury [22, 44]. Once blocked, the injury reduces [22, 44]. Hence, the study also indicates that CXR1/2 receptor stimulation has a positive effect on pulmonary recovery [41]. In this case, understanding its role in COVID-19 may contribute to clinical drug development. For patients suffering from ARDS, both CXCL8 and anti-CXCL8 levels increase which enhanced the survival of neutrophils [44]. CXCL8 plays a major role in the initial control of respiratory tract infection due to its chemotactic activity for neutrophils and monocytes [45]. And CXCL8 is considered a potential prognostic bio-marker for ARDS clinical course, which is a significant syndrome of SARS-CoV-2 [41].

In addition, this study has its limitations. The small sample size increases the error of the analysis results. If the analysis can be performed based on a large sample size, it may be possible to study the relationship between each central gene and the role of the pathway more fully so that the accuracy is better.

In conclusion, by studying the mRNA expression of human bronchial organoids, we have found that a series of DEGs (CXCL1, CCL5, EGF, CXCL10, CXCL8), which were also reported in SARS-CoV-2 infected patients, in vitro and in vivo models, plays an important role in the COVID-19 and are helpful for diagnosis and treatment. Our findings can better study the molecular mechanism of infection.

## Conclusion

In summary, using a series of biological analyses, we have obtained a new understanding and description of human bronchial organoids infected with novel coronavirus, and through the analysis and verification of differential genes, we have identified that CXCL1, CCL5, EGF, CXCL10, and CXCL8 play a key role in the infection process in novel coronavirus and a series of pathways related to cytokine expression, leukocyte migration, and cytokine-cytokine receptor interaction have been significantly changed. These findings will help study the infection mechanism and therapeutic control in novel coronavirus. In order for these biomarkers and targets to become a powerful tool for clinically diagnosing and studying the infection mechanism, more samples should be used to further study the expression differences of these genes. What is more, the pathways and genes mentioned above also play an important role in patients, in vitro and in vivo models infected with SARS-COV-2, and they may be a key link in the pathogenesis of COVID-19. Therefore, we preliminarily concluded that the bronchial organ model can be used as an in vitro model for the study of SARS-COV-2.

## Data Availability

The GSE150819 datasets used during the current study are available from GEO database (https://www.ncbi.nlm.nih.gov/gds/).
